# A switch from CD44^+^ cell to EMT cell drives the metastasis of prostate cancer

**DOI:** 10.18632/oncotarget.2841

**Published:** 2014-11-25

**Authors:** Zhiqun Shang, Qiliang Cai, Minghao Zhang, Shimiao Zhu, Yuan Ma, Libin Sun, Ning Jiang, Jing Tian, Xiaodan Niu, Jiatong Chen, Yinghao Sun, Yuanjie Niu

**Affiliations:** ^1^ Sex Hormone Research Center, Tianjin Institute of Urology, the Second Hospital of Tianjin Medical University, Tianjin, China; ^2^ University of Rochester, Rochester, New York, USA; ^3^ Department of Biochemistry and Molecular Biology, College of Life Sciences, Nankai university, Tianjin, China; ^4^ Department of Urology, Changhai Hospital of the Second Military Medical University, Shanghai, China

**Keywords:** androgen deprivation therapy, epithelial-mesenchymal transition, TGFβ1, cancer stem cell, CD44, prostate cancer

## Abstract

Epithelial–mesenchymal transition (EMT) has been linked to cancer stem-like (CD44+) cell in the prostate cancer (PCa) metastasis. However, the molecular mechanism remains elusive. Here, we found EMT contributed to metastasis in PCa patients failed in androgen deprivation therapy (ADT). Castration TRAMP model also proved PCa treated with ADT promoted EMT with increased CD44+ stem-like cells. Switched CD44+ cell to EMT cell is a key step for luminal PCa cell metastasis. Our results also suggested ADT might go through promoting TGFβ1-CD44 signaling to enhance swift to EMT. Targeting CD44 with salinomycin and siRNA could inhibit cell transition and decrease PCa invasion. Together, cancer stem-like (CD44+) cells could be the initiator cells of EMT modulated by TGFβ1-CD44 signaling. Combined therapy of ADT with anti-CD44 may become a new potential therapeutic approach to battle later stage PCa.

## INTRODUCTION

Epithelial–mesenchymal transition (EMT) has been shown to be a pivotal mechanism contributing to cancer invasion and metastasis [1], including prostate cancer [2], due to epithelial cells lose their polarity and acquire the migratory properties of mesenchymal cells in the developmental process. In recent researches on prostate cancer metastasis, EMT has been linked to stem cell phenotype [3, 4]. As reportedly, EMT results in the acquisition of stem cell-like properties including slow proliferation and self-renewal potential [5-8]. However, the molecular mechanism underlying EMT and regulation of stemness remains elusive. Early studies revealed EMT activators, such as Twist1, can co-induce EMT and stemness properties [3, 7], thereby linking the EMT and cancer stem cell concept [4]. However, metastasis is the process of invasive de-differentiated cancer cells leaving their primary tumor sites by EMT and colonized in a distant metastatic foci by re-differentiation (MET) [9]. Although many experimental reports fostered the concept of transient EMT-MET switches in metastasis, there are few directly proofs or strong evidences [10, 11]. Two were published supporting that the role of an EMT in dissemination and the need of a MET for efficient metastasis [12, 13]. It is not clear whether cancer stem/progenitor cell plays a role in EMT-MET switches. However, we proved, in this paper, that the epithelial de-differentiation towards caner stem/progenitor cells take place before EMT transition as an essential condition.

CD44, a major adhesion molecule of the extracellular matrix, being a marker of CSC has been identified positive expression in many tumors, either individually or in combination with other markers [14-18]. Recent study found that the microRNA-34a inhibited PCa caner stem/progenitor cells and metastasis by directly repressing CD44 [19]. Cyclin-dependent kinase-like 2 (CDKL2) as a novel potent promoter for EMT and breast cancer progression. CDKL2-expressing human mammary gland epithelial cells displayed enhanced mesenchymal traits and stem cell like phenotypes, which was acquired through activating a ZEB1/E-cadherin/β-catenin positive feedback loop and regulating CD44 mRNA alternative splicing to promote conversion of CD24^high^ cells to CD44^high^ cells[20]. Another published paper also showed that CD44s regulated the TGFβ-mesenchymal phenotype and was associated with poor prognosis in patients with hepatocellular carcinoma [21]. Therefore, we hypothesize that CD44 may play an important role in inducing EMT or in maintaining the mesenchymal phenotype in PCa.

It is well established that TGFβ_1_ plays important roles both in cancer EMT and cancer stem cell (CSC) properties [22], and is up-regulated in PCa after androgen deprivation therapy (ADT) [23, 24]. ADT is the standard therapy for advanced PCa since androgen ablation induces programmed cell death in malignant prostatic epithelial cells [25, 26]. Although TGF-β_1_ signaling is a major regulator of EMT and it helps to maintain the mesenchymal phenotype and stem cell states in an autocrine fashion in cancer [27]. However, a molecular linkage of this pathway that integrates the mesenchymal phenotype with the EMT process and CSC properties still remains unknown. Here, we assess the role of CD44 as a marker of cancer caner stem/progenitor cells in promoting EMT and matastasis in PCa carcinogenesis, and reveal a new potential therapeutic approach to battle PCa.

## RESULTS

### PCa patients treated with ADT develop more EMT

**Figure 1:**
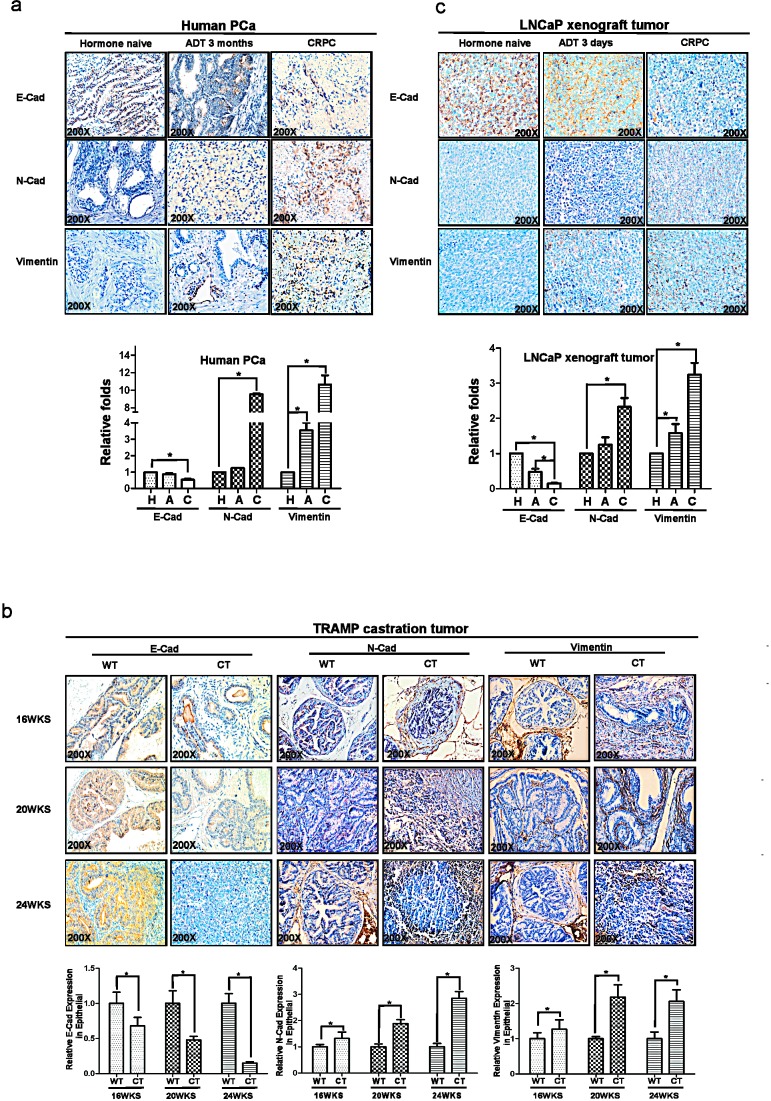
ADT promotes the development of epithelial-mesenchymal transition (EMT) in PCa tumors (a) Immunohistochemical (IHC) analyses the expression of EMT markers, E-Cadherin, N-Cadherin and Vimentin in human PCa samples hormone naïve, after 3month ADT and of CRPC specimens. (b) TRAMP mice were castrated at 12-wk-old, and then analyzed the expression of E-Cadherin, N-Cadherin and Vimentin in 16, 20, and 24-wk-old TRAMP PCa samples by IHC (c) LNCaP cells are orthotopically implanted into the anterior lobes of nude mice to generate the LNCaP xenograft tumors. LNCaP xenograft tumors supplied with DHT were treated as androgen sensitive controls, meanwhile samples from castration tumors (3 days) and from castration re-growth CRPC tumors were examined. The expression of E-Cadherin, N-Cadherin and Vimentin were detected by IHC. The histologic analyses of human PCa specimens, TRAMP mice samples and LNCaP xenograft samples were quantitated by image-pro plus 6.0 software as shown in fig1. Significance was defined as *p*<0.05(*).

We first compared PCa patients treated with ADT that already developed castration resistance (CRPC) vs those still sensitive to ADT treatment (ADPC) their EMT markers and found ADT-induced CRPC have altered EMT signaling with decreased E-Cadherin and increased N-cadherin and Vimentin (Fig.1a).

### TRAMP mouse PCa treated with ADT develop more EMT

We also applied TRAMP mouse model that can spontaneously developed PCa to metastasis to demonstrate the influence of ADT to PCa progression and altered EMT. We found PCa from TRAMP mice treated with ADT-castration expressed less E-Cadherin than those from WT-TRAMP mice in 20 wks and 24 wks (Fig. 1b). In contrast, higher expressed N-cadherin was found in the PCa from 20wks and 24wks TRAMP mice with ADT with castration than those from Wt-TRAMP mice (Fig. 1b).

### Orthotopically xenografted LNCaP mouse PCa treated with ADT develop more EMT

We then applied 2nd mouse model with orthotopically xenografted LNCaP cells that either sensitive to ADT or resistant to ADT treatment. We found that ADT resistant LNCaP xenografted PCa have lower E-Cadherin and higher N-cadherin and vimentin as compare to those ADT sensitive LNCaP xenografted PCa (Fig. 1c). Together, results from Fig 1a-c using either human clinical data or 2 different mouse models all demonstrated that ADT to target androgen/AR signaling led to promote EMT in PCa.

### Targeting androgen/AR signaling results in promoted EMT with alternation of cancer stem-like cell population

**Figure 2:**
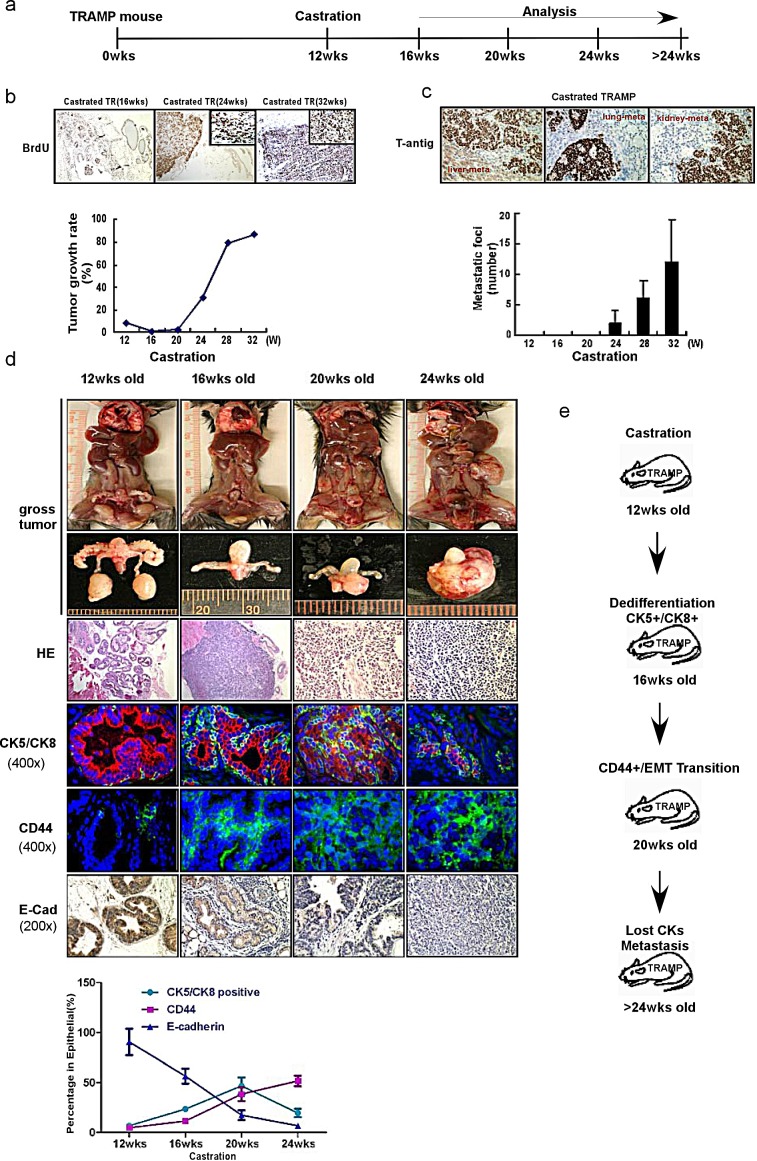
ADT sequentially alters the cancer stem-like cell populations and promotes EMT in TRAMP PCa tumors (a) The protocol of TRAMP mice experiments was illustrated. TRAMP mice were castrated at 12-week-old. Tumor samples were collected and analyzed at 12wks, 16wks, 20wks, 24wks and 32wks. (b) The growth rates of prostate epithelium were demonstrated by BrdU incorporation in TRAMP prostate before and after castration. The mice were i.p. injected with BrdU (10 ug/g body weight) every 6hr, and killed 24hr later. Paraffin-fixed tissue sections were stained by the BrdU detecting kit (Zymed Laboratories). The castration decreased the BrdU incorporation rates in 16wks-old mouse prostates, while the BrdU incorporation rates began to increase from 20wks and even higher than pre-castration from about 24wks (P<0.05), indicating the happen of CRPC. (c) Considerable metastatic foci in lung kidney and liver of were gradually increased from 24-wk-castration TRAMP mice. (d) From 20wks to 24wks, the castration resistant tumor continually grew bigger in gross (the first and second upper row). H&E staining showed that castration resistant tumors are poorly differentiated from 16wks to 24wks tumor samples (the third row). When double staining CK5 (green, marker for basal epithelial cell) and CK8 (red, marker for luminal epithelial cell) in immunofluorescence examination, the decreasing CK8^+^ staining following castration were detected, and the increasing CK5^+^/CK8^+^(yellow color, overlapped by green and red signals) cells, which indicated the expansion of intermediate cell population, were noticed in 16wks and 20wks tumors (the fourth row). Interestingly, the staining for epithelial markers, both CK5 and CK8, was significantly diminished in 24wks tumors. The CD44 expression significantly increased in 20wks and 24wks of castration resistant tumors (the fifth row). And the expression of E-Cadherin significantly decreased in 20wks and 24wks of castration resistant tumors (the last row). Significance was defined as *p*<0.05(*). (e) To summarize the sequential events following castration. After the TRAMP mice were castrated in 12wks, the dedifferentiation of epithelium, characterized by expanded CK5^+^/CK8^+^ cell population, happened in 16wks, followed by the EMT transition on 20wks and metastasis on 24wks.

In our TRAMP mouse model, mice were castrated at 12-weeks-old and tumor samples were collected at 16wks, 20 wks, 24wks, 28wks (Fig. 2a). We found that the proliferation marker, BrdU (and therefore, the growth rate too) was gradually increased from 16 wks tumors treated with ADT-castration to 32 wks tumor treated with ADT-castration castration tumor (Fig. 2b). In contrast, we found enhanced metastasis with more and more metastatic foci in liver, lung and kidney emerged from 24 wks tumor treated with ADT with castration (Fig. 2c).

To dissect the mechanism(s) why ADT will promote PCa metastasis in TRAMP mouse model, we found that epithelial marker E-Cadherin decreased with PCa progression after ADT-castration. Interestingly, we also found stem-like cell markers, such as CD44^+^ and CK5^+^/CK8^+^, increased with PCa progression after ADT-castration (Fig. 2d). We also noticed the luminal epithelial cell tumor gradually reduced yet CD44^+^ and CK5^+^/CK8^+^ stem-like tumor gradually increased from the 16wks (Fig. 2d), suggesting that while 16-20 wks tumor volume became smaller with decreased epithelial markers than those of 12 wks before ADT-castration, they did have increased stem-like cell markers with more metastatic foci formation (Supplemental Fig. 1). IHC staining also confirmed those cells in the metastatic foci were mainly composed of the cells with high cancer stem-like cell markers (Supplemental Fig. 1).

Together, results from Fig. 2a-d and supplemental Fig. 1 concluded that ADT with castration might promote PCa metastasis by inducing PCa cell EMT and alter the stem-like cell population.

### Activated TGFβ_1_ signaling lead to increase CD44+ stem-like cell population in two PCa cells

**Figure 3:**
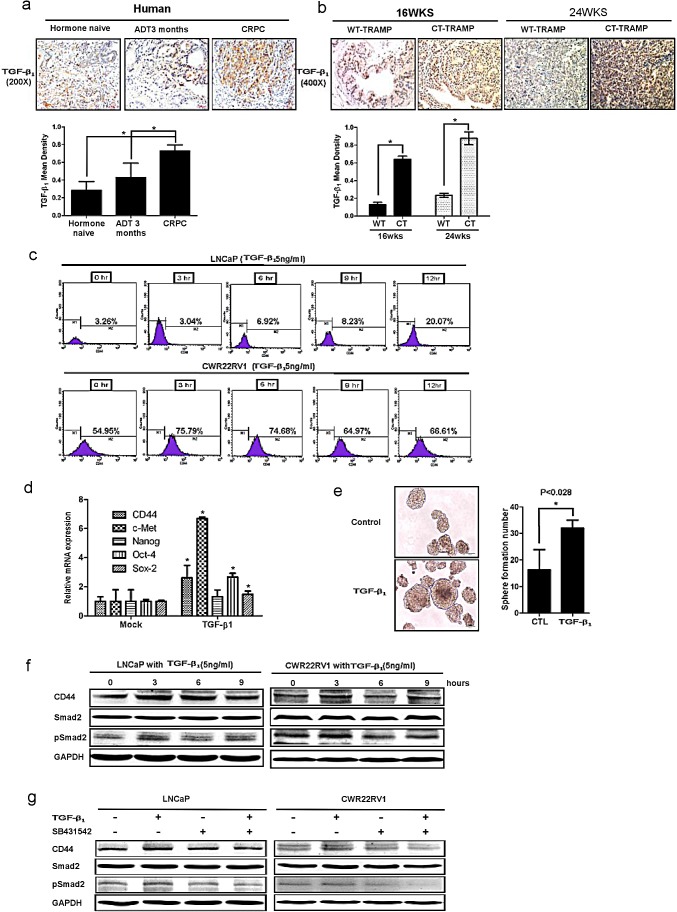
TGFβ1 can activate the dedifferentiation of PCa cells, leading to increased CD44+ S/P cell population (a) TGFβ1 was detected by IHC in human PCa samples before ADT, after ADT for 3 month, and of CRPC specimens. TGFβ_1_ expression was increased after ADT therapy. (b) TGFβ1 and phospho-Smad2/3 expression were increased in 16wks and 24wks castrated TRAMP prostate tumors comparing to wt TRAMP tumors. (c) TGFβ_1_ could expanse CD44^+^ cell population *in vitro*. LNCaP cells and CWR22rv1 cells were treated by 5ng/ml TGFβ_1_ for 3, 6, 9, and 12hrs. The CD44^+^ cell population was separated by flow cytometry. (d) 5ng/ml TGFβ_1_ increased the expression of cancer stem-like cell markers, including CD44, Oct-4, c-met, nanog and sox2 in LNCaP cells by real-time PCR assay. Data are in triplicate from three independent experiments and were normalized to GAPDH. All data are expressed as mean±S.D. (e) 5ng/ml TGFβ_1_ could also induce more sphere formation the character of S/P cells, compared to non-treatment LNCaP cells. Quantitation of the numbers of spheres (diameter>40μm) are presented as the mean *SD* (*Scale bar,* 100μm). (f) 5ng/ml TGFβ_1_ could up-regulate the expression of CD44 in LNCaP and CWR22RV1 cells via Western blot assay.(g) Importantly, We also applied interruption approach via using SB431542 to block TGFβ_1_ signaling and found CD44 expression decreased in Western blot assay. Significance was defined as *p*<0.05(*).

Previously studies found that activated AR might suppress TGFβ_1_ expression at transcriptional regulation, and ADT might lead to increased expression of TGFβ_1_ and TGFβ_1_ receptors plus its downstream Smad3. Here we found TGFβ_1_ signaling could also go through modulation EMT signaling and alternation the cancer stem-like cell population. As shown in Fig. 3c, TGFβ_1_ could enhance CD44^+^ cell population (via flow cytometry assay) in LNCaP and CWR22RV1 cells. Furthermore, TGFβ_1_ also increased cancer stem-like cell markers expression in LNCaP cells including CD44, Oct-4, c-met, nanog, sox2, and induced more sphere formation (that represent cancer stem-like cell formation) compared to wide type LNCaP cells (Fig. 3d&3e). Interestingly, TGFβ_1_ could also up-regulate cancer stem-like cell marker CD44 expression (via Western blot assay). Importantly, we also applied interruption approach via using SB431542 to block TGFβ_1_ signaling and found CD44 expression decreased by Western blot (Fig. 3f&3g).

Together, results from Fig. 3a-f concluded that ADT could enhance TGFβ_1_ signaling that might be able to increase CD44^+^ cancer stem-like cell population and expression of CD44 in PCa.

### TGFβ_1_ altered EMT with enhanced PCa invasion via modulation CD44

**Figure 4:**
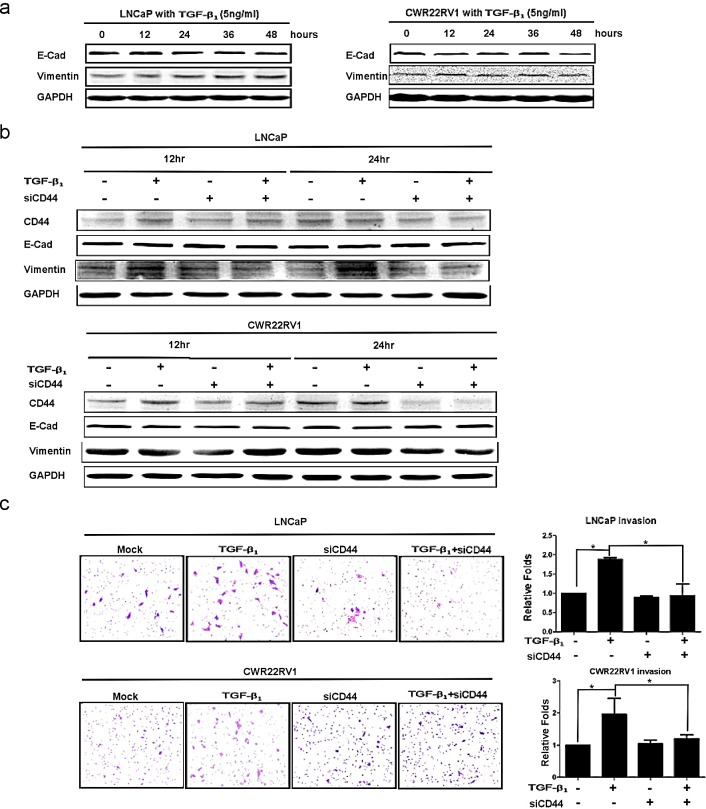
TGFβ1 altered EMT with enhanced PCa invasion via modulating CD44 (a) After 5ng/ml TGFβ_1_ treatment, the decrease expression of E-Cadherin and the increase expression of vimentin were detected in LNCaP and CWR22rv1 cells in a time dependent manner by Western blot. (b) EMT transition induced by TGFβ_1_ could be blocked by CD44 siRNA in LNCaP and CWR22rv1 cells. LNCaP and CWR22rv1 cells were treated with 5ng/ml TGFβ_1_ with or without CD44 siRNA for 12hrs and 24hrs. The expressions of CD44, E-Cadherin and Vimentin of different treatments were detected by Western blot assay. (c) Invasion ability of LNCaP and CWR22rv1 cells treated with TGFβ_1_ (5ng/ml), CD44 siRNA and both treatments were analyzed in Transwell Chamber assay. Quantitation was shown on the right. Significance was defined as *p*<0.05(*).

From above results, we demonstrated that TGFβ_1_ could alter CD44 expression and CD44^+^ stem-like cell population, we were interested to see if TGFβ_1_ signaling could modulate EMT and cell invasion via alternation of CD44 expression. We found that addition of 5ng/ml TGFβ_1_ led to EMT markers changed including increased vimentin and decreased E-Cadherin both in LNCaP and CWR22RV2 cell lines (Fig.4a). To confirm CD44 is a key regulating molecular in EMT induced by TGFβ_1_/pSmad2 signaling. We firstly found addition of 5ng/ml TGFβ_1_ led to increase the expression of CD44 and vimentin and decreased the expression of E-Cadherin in LNCaP and CWR22RV1 (Fig. 4b). We also found the invasion ability increased in LNCaP and CWR22RV1 cells with TGFβ1 treatment compared to those cells without TGFβ_1_ treatment (Fig. 4c). In contrast, TGFβ_1_ treatment in LNCaP and CWR22RV1 cells with knocked-down CD44 expression with CD44-siRNA led to little change in the expression of E-Cadherin (Fig. 4b) as well as the invasion ability of LNCaP and CWR22RV1 (Fig. 4c), suggesting TGFβ1 may need to go through modulation CD44 to alter the EMT and invasion ability of PCa.

### CD44^+^ caner stem/progenitor cells are responsible for mesenchymal transition and metastasis

**Figure 5:**
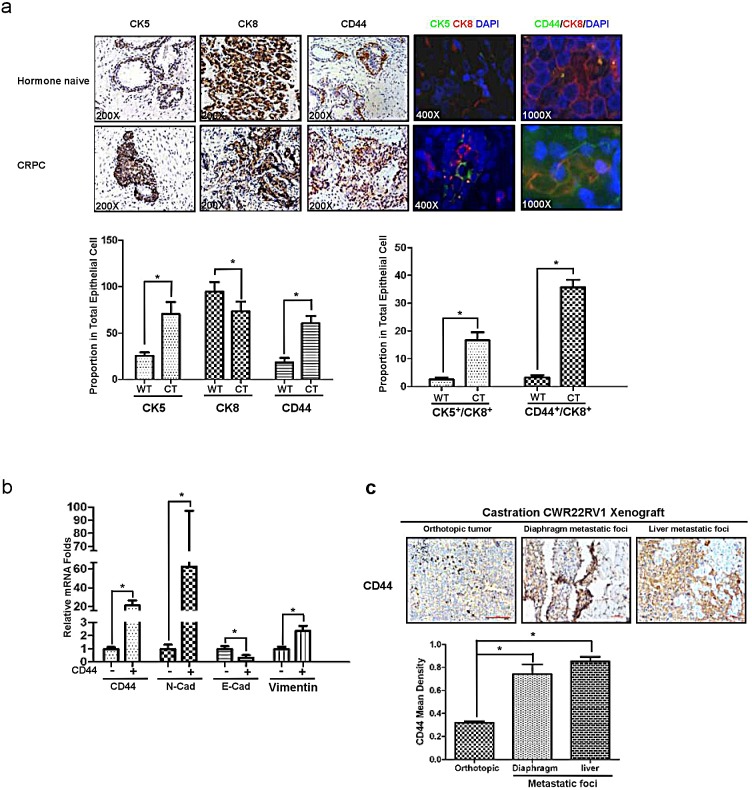
CD44^+^ stem-like cells are responsible for mesenchymal transition and metastasis (a) The increasing expression of CK5 and CD44 in human CRPC samples comparing with hormone naïve PCa was demonstrated in IHC staining (three lanes on the left), immunofluorescence double staining of CK5 and CK8(the fourth lane from left), and immunofluorescence double staining of CD44 and CK8 (the fifth lane from left). (b) CD44^+^ and CD44^−^ LNCaP cells were separated by MACS, their CD44, E-cadherin, N-cadherin and Vimentin expression were detected by real-time PCR. (c) CWR22rv1 cells were orthotopically implanted into the anterior lobes of nude mice prostate to generate xenograft tumors. CD44 expression in liver and diaphragm metastatic foci were compared to orthotopical xenograft tumors in IHC assay. Quantitation was shown in the right. Significance was defined as p<0.05(*).

Through the data from figure.5, we demonstrated that the increase of CK5 and CD44 expression were coincidently happened in CRPC samples comparing to those in human naïve specimens (Fig.5a, lane 1 and lane 3). And more importantly, CK5^+^/CK8^+^ (Fig.5a, lane 4) and CD44^+^/CK8^+^ (Fig.5a, lane 5) cells also increased in CRPC compared with those in hormone naïve. We further confirmed that CD44+ LNCaP cells expressed higher N-cadherin and Vimentin, with lower E-cadherin compared to those in CD44^−^ LNCaP cells (Fig.5b). Furthermore, we also identified high CD44 expression in liver and diaphragm metastatic tumors comparing to the primary tumor of CWR22rv1 orthotopic xenografts (Fig.5c). Together, data from Fig.5 suggested that EMT like cell might be originated from PCa luminal cell (CK8^+^), which de-differentiated to CD44^+^/CK8^+^ cell, then acquired properties of mesenchymal, such as high expression of N-cadherin and Vimentin, combined with low expression of E-cadherin. This kind of CD44^+^ cancer stem like cell could be as an initiator of EMT like cell with the function of metastasis. Therefore, targeting CD44^+^ cancer stem like cell may decrease PCa EMT and metastasis.

### Targeting CD44 led to decrease PCa EMT and metastasis *in vivo*

**Figure 6:**
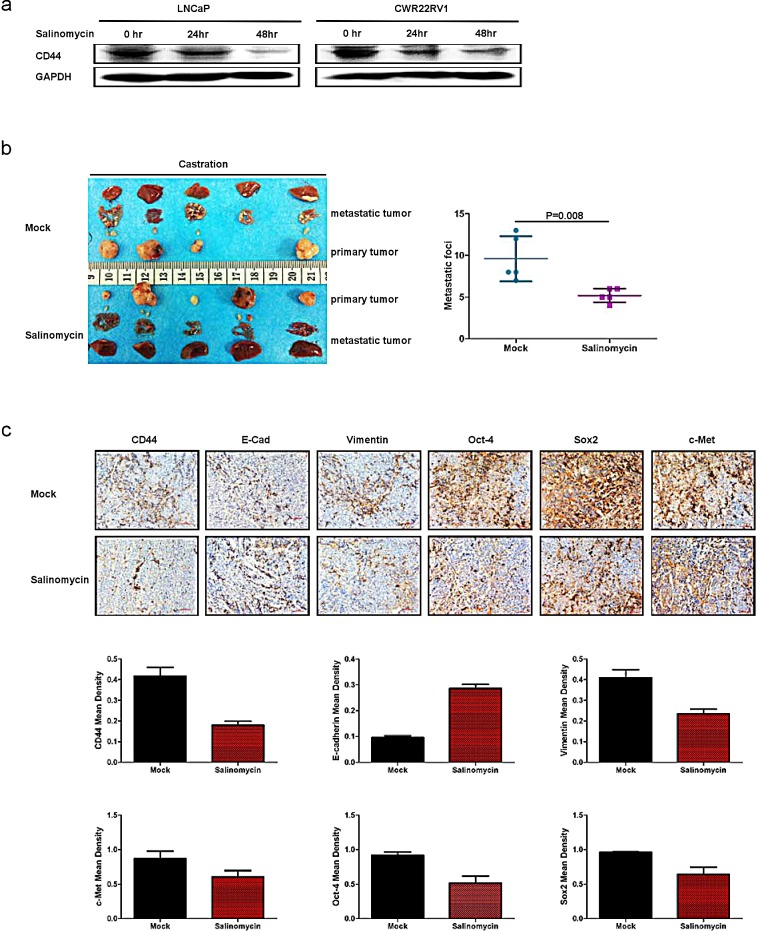
Targeting CD44 led to decrease PCa EMT and metastasis *in vivo* (a) Salinomycin can significantly suppress CD44 expression. CD44 expression was detected in LNCaP and CWR22RV1 cell lines treated with salinomycin by Western blot. (b) 5×10^6^CWR22rv1 were subcutaneously implanted on the dorsal of nude mice with androgen recruitment. After tumor diameter reached to 0.8cm, the tumor was harvested, divided into equal pieces and orthotopically transplanted into anterior prostates of either castrated or non-castrated nude mice. Each 5 from 10 castrated and 10 non castrated nude mice was treated with salinomycin by intraperitoneal injection, the others were intraperitoneally treated with corn oil as control. Metastatic tumors were found in salinomycin treatment groups as compared to those found from control groups. (c) The expressions of CD44, E-Cadherin, Vimentin, SOX2, c-Met and Oct-4 were analyzed by IHC. Quantitation was analyzed by image pro-plus 6.0 software. Significance was defined as *p*<0.05(*).

All above results suggest ADT to TGFβ_1_ to CD44 to EMT to PCa metastasis may represent a key signaling to influence the PCa metastasis. We are interested to see if target this newly identified signaling may yield any major impact on PCa metastasis. We then target CD44 with salinomycin to examine its therapeutic effect on the PCa progression. We first treated LNCaP and CWR22rv1 cell with salinomycin and found that salinomycin could decrease CD44 expression (Fig. 6a). We then applied CWR22rv1 xenograft mouse model via subcutaneously implanted 1×10^7^ CWR22rv1 cell on the dorsal prostate of nude mice. After tumor diameter reached to 0.8cm, it was divided into equal pieces and transplanted into the both prostate anterior lobe of castrated nude mice. One group of the mice were treated with salinomycin by intra-peritoneal injection, the others was intra-peritoneal injection with corn oil as control. The results showed the mice treated with salinomycin developed less malignant metastatic tumor as compared to those found from control group (Fig. 6b). Importantly, we also found decreased CD44 expression, with altered EMT markers for the decreased vimentin and increased E-Cadherin in the tumor with salinomycin treatment compared to those from control group (Fig. 6c). Furthermore, the expression of cancer stem cell markers like c-Met, Sox2, Oct-4 were also decreased in the tumor with salinomycin treatment compared to those from control group (Fig.6c).

Together, results from Fig. 6a-c concluded that targeting CD44^+^ cancer stem like cell led to decrease PCa EMT and metastasis *in vivo*.

### CD44 is a poor prognosis marker of PCa

**Figure 7:**
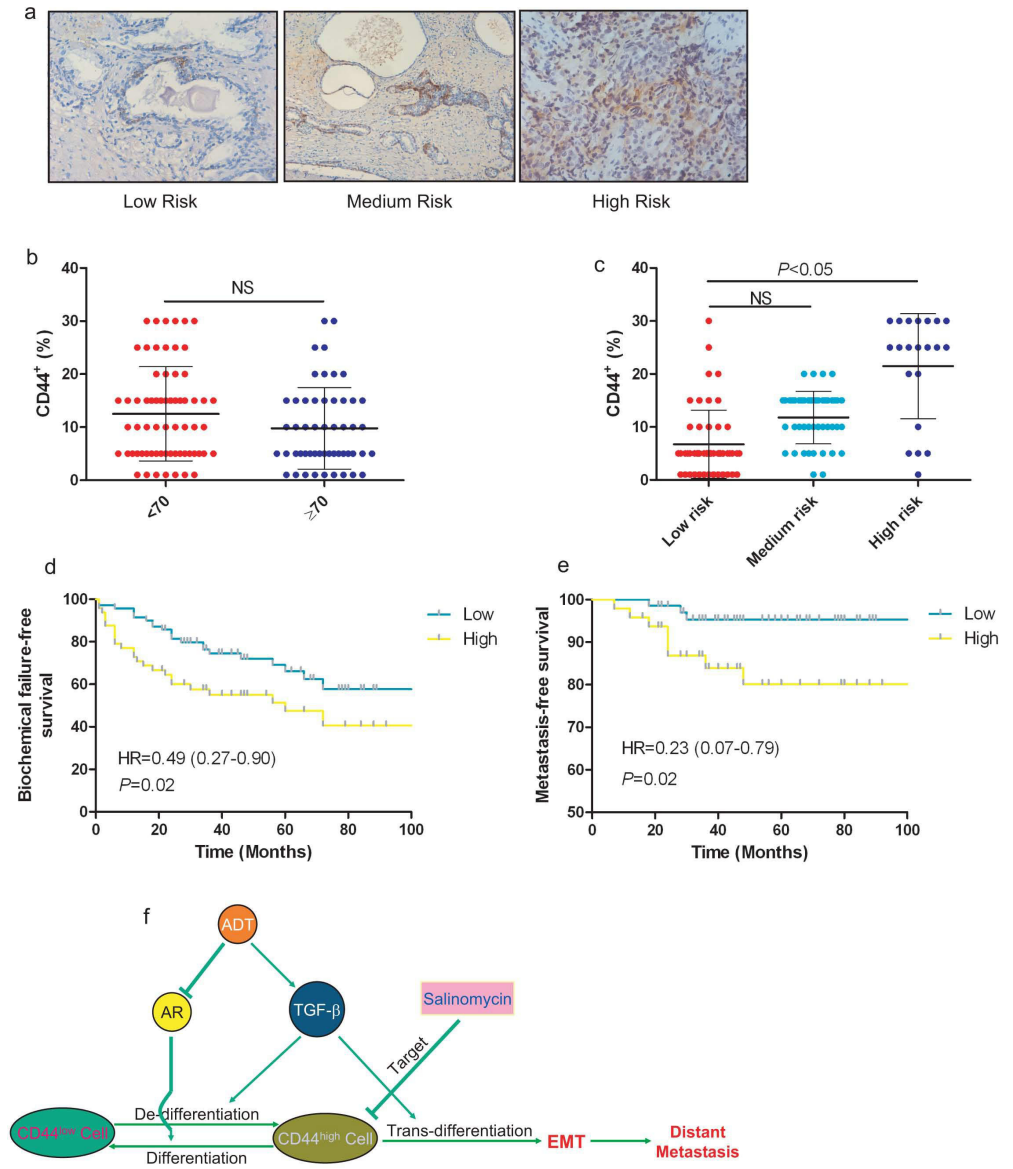
CD44 is a poor prognosis marker of localized PCa (a) CD44 expression in low-, medium-, and high-risk of human PCa was analyzed in IHC staining. (b &c) The expression levels of CD44 in 118 PCa patient samples were analyzed. A cut-off value of 10% was determined from the receiver operating characteristic (ROC) curve. Low and high CD44 staining were related to the disease risk but not age. (d & e) Kaplan-Meier curve analyzed the relationship between expression level of CD44 and biochemical recurrence or distant metastasis. Significance was defined as *p*<0.05(*) (f) Summarize the mechanism of ADT enhanced prostate cancer metastasis via alternation the TGFβ_1_-CD44 signaling.

Due to CD44^+^ cell population increased was one of essential conditions for EMT in CRPC, we further detected whether expression of CD44 was correlated with prognosis in clinic. The expression of CD44 was analyzed in 118 PCa patient samples. A cut-off value of 10% was determined from the receiver operating characteristic (ROC) curve, and according to this value, two groups (low and high CD44 staining) were assigned. The high level expression of CD44 was significantly related to the disease risk but not in age subgroup (Fig.7a-c). Kaplan-Meier curve was addressed to evaluate the role of CD44 as a prognosis marker, showing that high expression level of CD44 was associated with biochemical recurrence and distant metastasis (Fig.7d&7e). The above clinical data suggested that CD44 may become a poor prognosis marker of PCa.

We summarized the data and concluded that activation of TGFβ signaling induced by ADT led to de-differentiation phenotype as a result of increased CD44^+^ cancer stem like cell population, which subsequently generated to EMT like cell through TGFβ/CD44 signaling (Fig. 7f). Retarding PCa luminal cell de-differentiated to CD44^+^/CK8^+^ cell is a key step to control EMT and metastasis in PCa.

## DISCUSSION

Epithelial-mesenchymal transition (EMT), associated with prostate cancer progression, has given a reason to explain the altered expression of various lineage markers in pathological specimens. Although, cancer caner stem/progenitor cells has been suggested linked to EMT recently [7], the molecular mechanism involving the transition from caner stem/progenitor cells to mesenchymal phenotype cells remains elusive. Here, we demonstrate that CD44+ stem-like cell as an initiator of EMT cells contributes in cell transition and PCa metastasis, which is regulated by TGFβ_1_-CD44 signaling.

ADT challenge the hormone naïve as a stress, which forces the androgen dependent PCa cells death, while other cells, in response to survive pressure, become depending on other signaling instead of androgen signaling. In the prostate epithelial, there is a subtype of cells, so called basal or caner stem/progenitor cells, which are androgen insensitive. The dedifferentiation from luminal epithelial cells to basal or caner stem/progenitor cells has been reported previously [28]. Goldstein et al [29] has reported that the caner stem/progenitor cells exist in the luminal epithelial cells in the experimental condition of castration. In Fig.2, we show the phenomenon of the increase of CK5^+^/CK8^+^ double positive intermediate cells in 16wks castrated mice is the earlier event than EMT promotion till 20wks, indicating that the dedifferentiation of PCa cells is a critical step for EMT development. These changes in down-regulated epithelial markers and up-regulated mesenchymal markers resulted in the loss of prostatic glandular architecture and consistent with poor differentiated phenotype of aggressive PCa. Of note, in Fig.5, we found CD44^+^ cancer caner stem/progenitor cells results in EMT process rather than CD44^−^cells.

It is documented well that ADT increase the expression of TGF-β1 in PCa [24, 30], which induces EMT via many pathways including Smad or non-Smad-mediated pathways [31]. Interestingly, loss of the epithelium-specific transcription factor prostate-derived ETS factor (PDEF), which is down-regulated by TGF-β, induces EMT in PC3 cells, emphasizing the relationship between tumor de-differentiation and EMT [32]. Contrarily, prostate-derived ETS factor (PDEF) has been proposed as a tumor suppressor, could inhibit invasion and metastasis through reverse EMT [33]. Another study showed that maspin was sufficient to drive prostate tumor cells through a spectrum of temporally and spatially polarized cellular processes of re-differentiation, a reversal of EMT [34]. In this study, we show CD44, in response to TGFβ_1_, regulates the mesenchymal phenotype in prostate cancer cells. Our finding is consistent with another report that CD44^+^ prostate cancer cells are highly tumorigenic and metastatic [35].

Here, we target CD44+ cancer caner stem/progenitor cells with salinomycin resulting in suppress of EMT and metastasis, which has recently been documented to effectively eliminate caner stem/progenitor cells in different types of human cancers *in vitro* and in xenograft mice bearing human cancers [36-39]. Importantly, salinomycin is not only able to kill caner stem/progenitor cells, but also regular tumor cells and highly indolent tumor cells displaying resistance to cytotoxic drugs, radiation, and induction of apoptosis [40,41], may prevent prostate cancer metastasis. Due to ADT inducing EMT via stem-like differentiation, a potential therapy of targeting stem-like (CD44^+^) cells may suppress EMT, consequently, recurrence and metastasis in PCa. Therefore, targeting CD44^+^ cells, combined with ADT may become a new therapeutic approach to battle later stage PCa.

## METHODS

### Cell Culture and Reagents

CWR22rv1 (CRL-2505) and LNCaP cells were maintained in RPMI medium (Gibco) with 10% FBS (Gibco) at 37^o^C in 5% CO_2_. They were also supplemented with 1% Penicillin/Streptomycin. Purified recombinant human TGFβ_1_ (R&D Systems) was reconstituted in sterile 4 mmol/L HCl containing 1 mg/mL bovine serum albumin (Sigma). TGFβ_1_ was used at the indicated concentrations in serum-free medium. SB431542 were purchased from R&D Systems.

### Immunohistochemical (IHC) and Immunofluorescence (IF)

Basic IHC protocols have been described previously [42]. Primary antibodies for E-Cadherin (1:100 dilution; Abcam), vimentin (1:50 dilution; Abcam), N-cadherin (1:50 dilution; Abcam), cytokeratin 8 (CK8) (1:50 dilution, Abcam), cytokeratin 5 (CK5) (1:50 dilution, Abcam), CD44 (1:300 dilution; Abcam), TGFβ_1_ (1:100 dilution; Santa cruz) and phospho-Smad2 (1:100 dilution; Cell Signaling) were used for this study. Endogenous peroxidase activity was blocked using 3% hydrogen peroxide, and the sections were incubated with diluted antibodies. Slides were then incubated with various primary antibodies followed by Envision-plus labeled polymer-conjugated horseradish peroxidase and DAB monitoring staining (Zhongshan gold bridge, Beijing). To perform immunofluorescence experiments after deparaffinization, hydration and antigen retrieval as IHC protocol, slides were incubated with various primary antibodies followed by FITC or TRITC labeled second antibody and DAPI staining (Abcam). Finally, slides were viewed and imaged without dehydration.

### Protein extraction and Western blot analysis

Cells were lysed in RIPA buffer, separated on SDS-10% PAGE gel, and then transferred to a polyvinylidene difluoride membrane. After blocking by 5% nonfat milk and 5% FBS in PBST buffer, we immunoblotted the membrane with the primary antibody followed by incubation with HRP-linked secondary antibodies (GE Healthcare). The membranes were washed and visualized using a Chemiluminescent Detection Reagent Kit (ECL; GE Healthcare Corp.). Primary antibodies for E-Cadherin (1:1,000 dilution; Abcam), vimentin (1:1,000 dilution; Abcam), CD44s (1:1,000 dilution; Abcam), phospho-Smad2 (1:500 dilution; Cell Signaling), Smad2/3 (1:1,000 dilution; Cell Signaling), and GAPDH (1:1,000 dilution; cell signal) were used for this study.

### TRAMP Mouse and LNCaP Xenograft mouse model

TRAMP mouse were bought from Jackson lab and castrated at 12-wk-old. Tumor samples were collected and analyzed at 16wks, 20wks, 24wks and 28wks. Single cell suspensions of LNCap cell lines (5×10^6^ cells) in Matrigel (BD Biosciences) (1/1) were injected subcutaneously in the ﬂank of 8-wk-old BALB/C nude mice with androgen recruitment (Jackson Laboratories). Tumor incidence and growth was monitored during different time periods post injection. Tumors grew up to 1.0 cm in diameter, at which point animals were euthanized. After the diameter of LNCaP xenograft tumors reached to 0.8-1.0 cm, the tumor samples were collected and named androgen dependent xenograft tumor. We removed androgen supplementary from the nude mice with diameter-0.8cm LNCaP xenograft tumors and castrated them for one week, then sacrificed the mice and collected tumors named ADT tumor. When the diameter of LNCaP xenograft tumor re-grew to 0.8cm in the castrated mice, we sacrificed the mice and collected the tumors named castration resistant LNCaP xenograft tumor. Each tumor was dissected, ﬁxed in formalin, and processed for histopathology examination.

### Orthotopic xenograft

Single cell suspensions of CWR22rv1 cell lines (5×10^6^ cells) in Matrigel (BD Biosciences) (1/1) were injected subcutaneously in the dorsal of 8-wk-old BALB/C nude mice with androgen recruitment (Jackson Laboratories). After tumor diameter reached to 0.8cm, the tumors were divided into equal patches and orthotopically implanted them into the both prostate anterior lobe of 8-wk-old BALB/C nude mice, which were castrated one week before. They were divided into two groups. Each group has 5 mice. One group were treated with salinomycin by intraperitoneal injection, the other group were intraperitoneally treated with corn oil as control. Mice were killed 14 wks later, and orthotopic xenograft and metastatic tumors were fixed and embedded in paraffin for further analyses.

### RNA extraction and Real-time RT-PCR

RNA was isolated using the RNAprep Pure Cell/Bacteria Kit (TianGen, Beijing, China) according to the manufacturer's instructions and digested with DNase I to prevent amplification of genomic DNA. Reversed transcription was performed using Quant Script RT Kit (TianGen, Beijing, China). The 20μl cDNA was then diluted by water into 200μl. 2μl reverse transcribed cDNA were used for PCR and real-time quantitative PCR the MyCycler thermal cycler (Bio-RAD) with by Taq polymerase and on the iCycler IQ multicolor real-time PCR detection system with 1/5μl cDNA amplified by SYBR Green PCR Master Mix, respectively. The PCR amplification conditions were as follows: pre-denaturing at 95C for 3 min, followed by 40 cycles of amplifications by denaturing at 95°C for 30 s, annealing at 60°C for 1 min, extension at 72°C for 1 min. After a final extension at 72°C for 10 min, the amplified products were subjected to a stepwise increase in temperature from 55 to 95°C to construct dissociation curves. Each sample was run and analyzed in triplicate.

### BrdU Incorporation Assay

We purchased 5′-Bromo-2′-deoxyuridine (BrdU) from Sigma and dissolved it in double distilled water at 10 mg/ml. Starting at 24hr before sacrifice, we injected mice i.p. every 6h with 10 μg BrdU per gram body weight. Following harvest, we embedded tissues in paraffin and labeled them following the BrdU Staining Kit (Zymed) manufacturer's instructions.

### Flow Cytometry

We digested the cells by trypsin-EDTA, harvested as many as 1×10^6^ cells, and fixed them in 70% ethanol at 4°C. After 12h, cells were centrifuged (1,000×g, 5min, 4°C), resuspended in PBS containing 0.05 mg/ml Rnase A (Sigma), and then incubated with CD44-PE (Sanjian, Tianjin, China) at room temperature for 30 min. After washing and staining with 10 mg/ml propidium iodide, cells were filtered through a 60-mm mesh, and 10,000 cells were analyzed by flow cytometry (FACS Calibur, BD Company) with ModFit software (Verity Software House, Inc.).

### Migration and Invasion Assays

To analysis invasion, CWR22rv1 and LNCaP cells were treated with TGFβ_1_; after 48hrs, 2.5 × 10^4^ cells were seeded into 24-well cell culture inserts with 8 μm pores (BD Falcon). As a chemoattractant, 10% FBS was used in the lower chamber. Cultures were maintained for 48 h, then non-motile cells at the top of the ﬁlter were removed and the cells in the lower chamber were ﬁxed with methanol and stained with Diff-Quick staining kit (Dade Behring). Either the number of cells per well or ﬁve different ﬁelds per condition was counted by microscopy. Relative invasion was calculated in relation to the control.

### Mammosphere and Tumor sphere Assay

Assays were performed as previously described with modiﬁcation [43]. 1000 cells/well were seeded in 6-well ultra-low adhesion plates (Costar) in MEGM medium containing 10% Serum Replacement (KnockOut™) supplemented with 1× MEM non-essential amino acid (Gibco), 20 ng/ml EGF (R&D Systems), 10 ng/ml bFGF (R&D Systems), and B27 (GIBCO). For secondary sphere formation, primary spheres were dissociated by trypsinization and replated at 1000 cells/well.

### Clinical PCa Patients Study

A total of 118 cases of primary PCa diagnosed between 2003 and 2009 were retrieved from the medical files of the 2nd Hospital of Tianjin Medical University, China. The median follow-up was 45 months ranged from 9 to 120 months. The study was conducted under a protocol approved by Institutional Review Board of the 2nd Hospital of Tianjin Medical University. Of the 118 patients, 46 had biochemical recurrence before 72 months (median time to recurrence 21 months, ranged from 3 to 72) and 72 were free of disease recurrence (mean follow-up 48 months, ranged from 21 to 120). Biochemical recurrence was deﬁned as a PSA level > 0.2 ng/ml in 2 successive measurements (ie, two consecutive double-fold increases) after radical prostatectomy [44]. Patient characteristics including age, preoperative PSA (iPSA) and tumor pathology (i.e., Gleason sum, stage, margin and seminal vesicle invasion) are collected, and then disease risk was classified according to guideline from European Association of Urology (EAU)[44] based on above characteristics. In brief, the risk patterns were classified into three groups: 1) low-risk PCa (iPSA < 10 ng / ml and Gleason score≤6 and cT1c–cT2a); 2) intermediate-risk PCa (PSA 10.1-20 ng / ml or Gleason score=3+4 or cT2b-c); 3) high-risk PCa (PSA > 20 ng / ml or Gleason score≥4+3 or ≥cT3a).

### Scoring of CD44 Expression

After IHC staining, the entire section was evaluated by two respective observers being unaware of the clinical data. Slides for CD44 were scored like described by Ekici et al [46]. The tumor cells were determined positive when a clearly visible staining signal was detected on the cell membrane. Normal prostate tissue and/or benign prostatic hyperplasia glands were included to be as internal controls. Positive cell was graded as 1%, 5%, 10%, 15%, 20%, 25% and 30%. CD44 positive basal cells in benign para-carcinoma tissue were not included during the scoring process. A cut-off value was determined from the receiver operating characteristic (ROC) curve, and according to this value, 2 groups (low and high CD44 staining) were assigned.

### Statistics

Data are presented as mean ± SD unless otherwise indicated. A Student's t test (two-tailed) was used to compare two groups (p < 0.05 was considered signiﬁcant) unless otherwise indicated. Survival curves were analyzed by Kaplan-Meier analysis and log-rank tests.

## SUPPLEMENTARY MATERIAL AND FIGURE


