# Supplementary material for: A switch from CD44+ cell to EMT cell drives the metastasis of prostate cancer
Source: Oncotarget. 2014 Nov 25;6(2):1202–16. doi: 10.18632/oncotarget.2841 (PMC4359227; doi:10.18632/oncotarget.2841)
Supplement: Supplementary file 1 [file oncotarget-06-1202-s001.pdf]

A switch from CD44^{+} cell to EMT cell drives the metastasis of prostate cancer

Supplementary Material

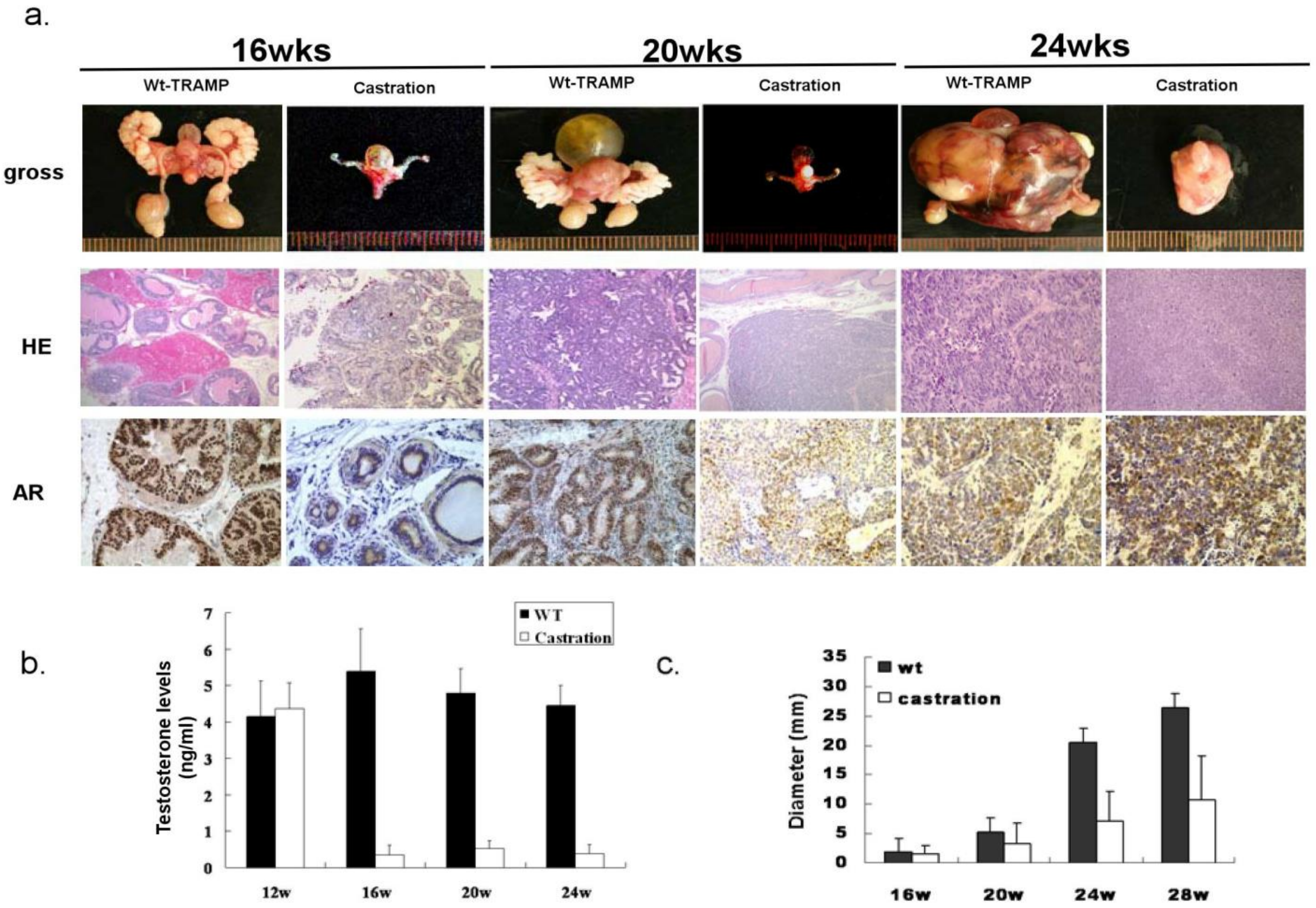

Supplemental Figure 1
